# Radiation-Induced Organizing Pneumonia: A Characteristic Disease that Requires Symptom-Oriented Management

**DOI:** 10.3390/ijms18020281

**Published:** 2017-01-27

**Authors:** Keisuke Otani, Yuji Seo, Kazuhiko Ogawa

**Affiliations:** Department of Radiation Oncology, Graduate School of Medicine, Osaka University, Suita 565-0871, Japan; seo@radonc.med.osaka-u.ac.jp (Y.S.); kogawa@radonc.med.osaka-u.ac.jp (K.O.)

**Keywords:** organizing pneumonia, bronchiolitis obliterans organizing pneumonia, breast cancer, corticosteroid treatment, radiation-induced organizing pneumonia

## Abstract

Radiation-induced organizing pneumonia (RIOP) is an inflammatory lung disease that is occasionally observed after irradiation to the breast. It is a type of secondary organizing pneumonia that is characterized by infiltrates outside the irradiated volume that are sometimes migratory. Corticosteroids work acutely, but relapse of pneumonia is often experienced. Management of RIOP should simply be symptom-oriented, and the use of corticosteroids should be limited to severe symptoms from the perspective not only of cost-effectiveness but also of cancer treatment. Once steroid therapy is started, it takes a long time to stop it due to frequent relapses. We review RIOP from the perspective of its diagnosis, epidemiology, molecular pathogenesis, and patient management.

## 1. Introduction

Pneumonia is one of the most common causes of death around the world, but various pathogeneses may be responsible. It is divided into alveolar and interstitial pneumonia, and interstitial pneumonia needs further classification [1]. Organizing pneumonia (OP) is a type of interstitial pneumonia and consists of cryptogenic organizing pneumonia (COP) and secondary organizing pneumonia (SOP) [2]. Radiation-induced organizing pneumonia (RIOP), which also used to be called radiation-induced bronchiolitis obliterans organizing pneumonia (BOOP) syndrome is one type of SOP. It was first reported by Crestani et al. and Bayle et al. in 1995 [3,4]. Since the term “bronchiolitis obliterans” does not reflect the actual pathophysiology, the name BOOP was replaced by organizing pneumonia. According to this, the name radiation-induced organizing pneumonia (RIOP) is considered better [5]. Several reports of RIOP were published, and its incidence was reported to be less than 3% [5,6,7,8,9,10,11,12] (1.7% in an extensive literature review [13]) after radiotherapy involving the breast. Although RIOP is classified as SOP, no actual fatal cases have been reported so far. However, the mortality of OP is estimated to be 5% [14], and SOP is reported to have a higher mortality than COP [15,16], so physicians need to pay attention in the management of RIOP.

## 2. Diagnosis

In the report of Crestani et al., the following four criteria were introduced [17]: (1) radiation therapy to the breast within 12 months; (2) general and/or respiratory symptoms lasting for at least two weeks; (3) lung infiltrates outside the radiation port; and (4) no specific cause. However, subsequent reports contained patients who did not fulfill all of these criteria, and the nature of RIOP was gradually revealed, which requires us to reconsider them.

### 2.1. Partial Irradiation Involving the Lung Occurring Approximately within 12 Months

Since most reports were based on the original criteria, most of the RIOP cases in the literature were diagnosed within 12 months after the completion of radiotherapy. Among them, up to 90% of the reported cases were diagnosed within six months after the completion of radiotherapy [5,6,7,8,9,11,18,19,20]. However, RIOP cases after more than 12 months were also reported; Arbetter et al. reported one case after 17 months, who was diagnosed by resection of an asymptomatic lung nodule [19]; Kubo reported a case after 23 months whose diagnosis was confirmed by bronchoscopy and whose onset was presumed to be delayed by immunosuppressive agents prescribed for coincident rheumatoid arthritis [9].

RIOP is not specific to irradiation of the breast or to women. Although reports are limited, RIOP has also been reported in lung cancer and thymoma patients, including male patients [21,22,23,24,25]. This fact supports the idea that the pathogenesis of RIOP is not related to breast cancer or sex, but to partial irradiation involving the lung. In addition, not only the conventional irradiation technique, which takes more than one month, but also ablative radiotherapy, which is completed within one or two weeks, can cause RIOP. Murai et al. and Ochiai et al. reported RIOP after stereotactic body radiotherapy (SBRT) for lung tumors [26,27]. Interestingly, the onset is longer after SBRT than after conventional radiotherapy; it has been reported to be at least 6 months after completion of radiotherapy and sometimes more than 12 months, in contrast to the short duration of treatment.

### 2.2. Lung Infiltrates Outside the High Irradiation Dose Area

After the completion of radiotherapy involving the lung, radiographic pulmonary changes on computed tomography (CT) are reported in 78% of cases at 3–9 months after radiotherapy and are thus common [28]. In the management of breast cancer patients, the volume of these changes is usually limited and seldom causes symptoms in this range. RIOP is noticed by the spreading of infiltrate outside the irradiated volume [3,4]. Chest radiographs show air-space opacities or diffuse infiltrative opacities, which are often detectable without CT scans. On CT scan images, peripheral air-space opacities with air bronchograms and ground-glass opacities are common, and multiple alveolar opacities on imaging represent the most frequent and typical imaging features of OP [29]. Migratory infiltrates are another feature of RIOP [4,5] and can be observed until corticosteroid therapy is initiated [17]. Ogo et al. classified the infiltrative pattern into four types: Type A, peripheral area in the radiation field and a continuous opacity that represents consolidation with or without an air-bronchogram, ground-glass attenuation, and/or nodular opacity; Type B, peripheral area in the radiation field and continuous alveolar infiltration in the zone of the middle lung; Type C, peripheral area in the radiation field and isolated consolidation on the back side of the radiation field; and Type D, peripheral area in the radiation field and consolidation or ground-glass attenuation (or both) in the contralateral side [8]. Among these patterns, Type A was the most common (65%), and multiple types were observed in 43% of patients. The clinical course of lung infiltrates was also classified into 3 types: Type 1, the ipsilateral side; Type 2, progressing on the ipsilateral side; and Type 3, moving from the ipsilateral side to the contralateral side [8]. Type 2 (13%) and Type 3 (22%) would correspond to migratory lesions in other reports [4], but their incidence varies from 17% to 100% [5,7,17,19,20].

### 2.3. No Other Specific Cause

Organizing pneumonia is known to occur from several triggers. The most important cause to be distinguished is infection, since subsequent corticosteroid treatment might worsen the condition. Bacterial pneumonia sometimes resembles OP on radiographic examination. Microbial culture of sputum or bronchoalveolar lavage (BAL) fluid would be helpful in ruling out bacterial infection. When BAL fluid is available, it shows an increase in lymphocytes, mast cells, CD3 cells, and CD8 cells, and a decrease in CD4 cells and the CD4/CD8 ratio [30], and this would support the diagnosis of RIOP.

Cottin et al. reported that chronic eosinophilic pneumonia (CEP) occurred after radiotherapy to the breast [31]. Interestingly, the clinical features of CEP after radiotherapy were almost the same as those of RIOP: CEP was observed 1–10 months after completion of radiotherapy; the migratory lung infiltrate was also similar to that of RIOP; and corticosteroids worked well. Considering these similarities, patients with CEP after radiotherapy might have been diagnosed as RIOP. Since all patients had a history of asthma or atopy [31], patients with allergy seemed to be predisposed to CEP. Eosinophilia may be a key finding to discriminate it from RIOP, and an increased number of eosinophils in the BAL fluid may support the diagnosis. However, considering that corticosteroid works effectively, as in RIOP, as mentioned above, definite discrimination of these two would not be critical in managing patients.

General and/or respiratory symptoms lasting for at least two weeks were another diagnostic criterion. Clinical manifestations include fever, nonproductive cough, dyspnea, malaise, fatigue, chest pain, and weight loss [8,13,17]. However, several reports included asymptomatic patients [5,10,19] who manifested similar radiographic features or histopathologic observations. RIOP has gradually gained attention, and the diagnosis of RIOP can often be made within two weeks. Despite the impressive lung imaging findings, symptoms are not always serious and can be relieved by antitussives or non-steroidal anti-inflammatory drugs within two weeks. Indeed, the symptoms are important when considering the management of RIOP, but the diagnosis of the massive lung infiltrates does not require the presence of symptoms.

Although these criteria describe the features of RIOP, RIOP is ultimately COP with a history of irradiation. In the current criteria, RIOP includes genuine COP after radiotherapy by chance. It is not very important to discriminate them because their general management is similar [14], but we should carefully diagnose RIOP as much as COP if the patient’s general condition is unfavorable. If the treatment response is poor, or the clinical course or chest imaging findings are not typical for RIOP, a video-assisted thoracoscopic lung biopsy should be considered to confirm a definitive diagnosis of OP [13].

## 3. Pathogenesis and Epidemiology

OP is known as an inflammatory response to acute lung injuries. Development of OP has been reported to go through three stages [32]. The first stage is the injury phase: local epithelial injury induces the death of pneumocytes and the formation of gaps in the basal lamina [29,33]. The second stage is the proliferating phase: fibroblasts and inflammatory cells, such as lymphocytes, neutrophils, and eosinophils, infiltrate the alveolar interstitium and form fibroinflammatory buds. Activated fibroblasts proliferate, differentiate into myofibroblasts, and form cell clusters within the distal airspaces. The third phase is the mature phase: mature fibrotic buds occupy the lumens of bronchioles, alveolar ducts, and adjacent peribronchiolar alveoli [29,33,34]. Recently, transgenic mice overexpressing human C-C motif chemokine ligand 2 (hCCL2) under control of the surfactant protein C promoter in type II alveolar epithelial cells were reported to work as an animal model for OP [35]. This animal model showed pathogenomic, molecular, and morphological features of human OP and exhibited a similar inflammatory profile, which is a key feature in interpreting human OP. In this model, the generation of OP was completed in 7 days. As for RIOP, the epithelial injury would be irradiation-induced. The most lethal effect in the irradiated cells is DNA double-strand breaks, and the cells in the thorax develop apoptosis if they fail to recover from the DNA damage. Interestingly, cell death after irradiation can start within 10 h [36], but the onset of RIOP is delayed by about 6 months after the completion of radiotherapy. Furthermore, RIOP after SBRT, which delivers a higher dose to the cancer and circumscribed lung tissue than does conventional radiotherapy to the breast, is delayed even more [26,27]. In COP cases, the time to onset is known to be less than three months [32]. Additional insights are necessary to understand what is going on during this long gap. When we look into other secondary OPs, drug-related OP can occur months to years after drug administration [14]. The first step to treating drug-related OP is to discontinue the suspected agents. In contrast, most cases of RIOP occur after completion of radiotherapy. The trigger had already been disseminated and already finished several months before the onset of RIOP.

As another explanation of the pathogenesis of RIOP, mutations in the ATM (ataxia telangiectasia mutated) gene were proposed [37]. The ATM gene is a key molecule to repair DNA double-strand breaks whose mutation brings hypersensitivity to irradiation [38] and predisposes to cancer [39]. A patient with RIOP who was also diagnosed to have monoallelic germline ATM mutation was reported [37]. Interestingly, the frequency of ATM mutation carriers among women affected with breast cancer has been estimated to be 2.04% [40], similar to that with RIOP. This might be the explanation of the long time between radiotherapy and RIOP. Further epidemiologic evidence and analysis of causal molecular background are expected.

Several risk factors have been identified for RIOP: age [11,41], irradiated lung volume [9], concurrent endocrinology [11], and smoking [13,41]. Kubo et al. reported that a central lung distance of more than 1.8 cm, which means the extent of irradiated lung volume is a risk for RIOP [9]. Considering the pathogenesis, it is reasonable that some extent of irradiated lung volume is required to prime the consequent immune-responses that lead to RIOP. However, a central lung distance of below 1.8 cm is too strict a limit to require for every breast-irradiation treatment. Since the incidence of RIOP is below 3.0% [13] and its prognosis is generally good, the radiation field should not be minimized too much for fear of RIOP.

As another risk factor for RIOP, concurrent endocrine therapy is controversial. Katayama et al. reported that endocrine therapy, including both tamoxifen and anastazole, was a risk factor [11], but Kubo did not find it to be significant [9]. Tamoxifen is known to induce transforming growth factor-β (TGF-β) secretion, which causes lung fibrosis [42,43]. Epidemiologic observations reported that tamoxifen was a risk for lung fibrosis on multivariate analysis [44]. However, epidemiologic data showed that concurrent usage of tamoxifen does not increase the incidence of pneumonitis compared to the sequential usage of tamoxifen [45,46]. Today, the concurrent usage of tamoxifen is considered tolerable. As for aromatase inhibitors, a randomized controlled trial showed that the incidence of lung fibrosis was similar between concurrent and sequential administration of aromatase inhibitors [47]. Although these data did not refer to RIOP as a specific disorder, concurrent endocrine treatment is considered tolerable. Because of the rarity of RIOP, it is difficult to assess the actual risk of concurrent endocrine therapy, but it seems that endocrine therapy need not be interrupted at the initiation of radiotherapy.

## 4. Patient Management

RIOP is generally a complication with a good prognosis due to the acute effectiveness of corticosteroids [17]. However, relapse of RIOP is common when steroids are administered [13,17]. Okada et al. reported that relapse after steroid therapy was associated with higher C-reactive protein levels at RIOP diagnosis [12]. We previously reported that steroid-treated patients relapsed at a significantly higher rate [5]. Since no new lesion was recognized during steroid administration, we assumed that steroid therapy effectively suppresses the development of new lesions (migratory infiltrate). However, this suppressive effect seemed to decline with steroid tapering; therefore, new lesions (relapses) appeared particularly in patients in the steroid group. We hypothesized two reasons: steroids can postpone the development of new lesions by suppressing tissue responses to inflammation; and they can cause the relapse of tissue-damaging responses.

We also showed that patients in the steroid group took less than half a month to achieve symptom relief after the administration of steroids. Other reports also indicate that steroid therapy has a strong role in the rapid relief of initial RIOP symptoms [6,17]. In contrast, some patients in the nonsteroid group experienced persistent symptoms for up to three months [5]. Since nonsteroidal treatment of RIOP was reported to result in almost normal lung function without severe sequelae [5,10], steroids can be saved as a last resort in managing RIOP. If RIOP is relieved without steroid therapy, the overall time to become free of symptoms, steroids, and other medications would be shorter than with steroid treatment [5]. Adverse effects of systemic steroid therapy include infection, adrenal insufficiency, osteoporosis, peptic ulcer disease, cataract formation, dermal thinning, hypertension, diabetes, psychosis, and hyperadrenocorticism [48], and additional management is necessary once steroid is initiated. Steroid is routinely used in several situations during cancer treatment, such as controlling nausea and vomiting and improving symptoms caused by advanced disease [49]. However, patients who undergo chemotherapy are predisposed to lung infections [50], and steroid therapy shares this risk. In addition, use of corticosteroids in patients with solid tumors could induce resistance to treatment in cancer cells in some patients [49,51,52]. Thus, nonsteroidal treatment is favored in terms not only of short treatment time, requiring less care and medicine, and therefore cost-effectiveness, but also of treatment effectiveness for cancer.

RIOP is often noticed during the follow-up after completion of radiotherapy. RIOP patients may come to clinics with flu-like symptoms or massive lung infiltrates. For the physicians who are not engaged in their cancer treatment, getting the history of radiotherapy is the first step to diagnosing RIOP. The next step is to evaluate the chest X-ray. As for breast cancer patients, lung infiltrates of RIOP are obvious, in contrast to radiation pneumonitis. If the chest X-ray is abnormal, CT is necessary. These steps are similar to common pneumonias. If there is a history of radiotherapy within one year or more, and infiltrates outside the high-dose irradiated area are confirmed, the diagnosis of RIOP takes just one more step, but it is the most difficult one. On the radiographic appearance, if typical imaging features of OP are seen, most experienced clinicians may make the diagnosis [29]. COP had the highest rate of correct diagnosis among the interstitial pneumonias, in 79% of cases [53], suggesting that the CT imaging features are characteristic. However, it is not always easy to rule out infection. Sputum culture is recommended if available. Empiric antibiotic therapy is permitted for ambiguous cases because it does no harm to RIOP, but it makes the diagnosis unclear. BAL is indicated in all cases where COP is suspected [29]. Since RIOP includes various general conditions, patients with no or mild symptoms should undergo repeated chest imaging instead of BAL [13]. Patients with severe symptoms and/or considerable steroid therapy will benefit from BAL fluid analysis.

**Figure 1:**
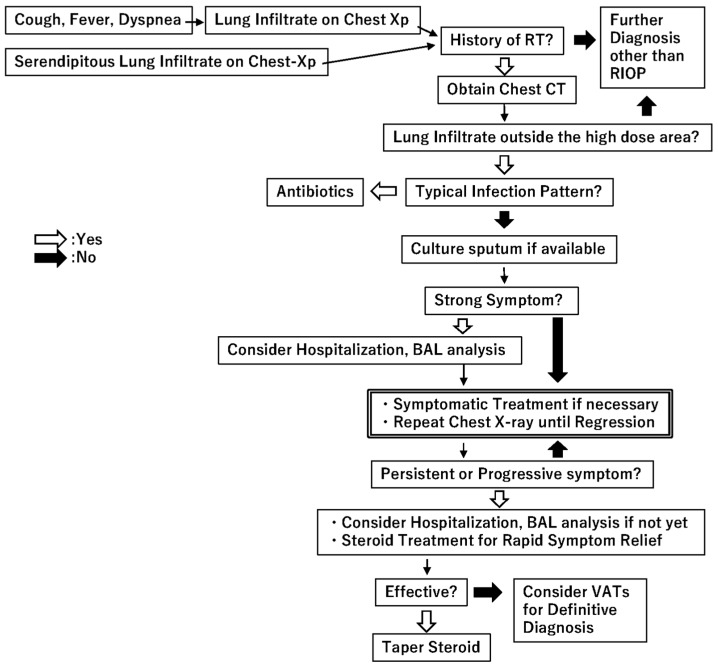
Diagnostic and treatment decision tree of radiation-induced organizing pneumonia. Abbreviations: RT: Radiotherapy; RIOP: radiation-induced organizing pneumonia; CT: computed tomography; BAL: bronchoalveolar lavage; VATs: video-assisted thoracoscopic lung biopsy.

Treatment of RIOP should simply be symptom-oriented (Figure 1); no medication is necessary for asymptomatic patients, and antitussives and non-steroidal anti-inflammatory drugs can be prescribed for patients with cough and/or fever. If the patient develops shortness of breath, evaluation of hypoxemia is necessary, and hospitalization should be considered depending on the degree. Steroid treatment can be used whenever rapid symptom relief is required, but it should be restricted to patients who have already undergone BAL fluid analysis and the diagnosis of RIOP is supported. If steroid treatment response is poor, or the clinical course or chest imaging findings are not typical for RIOP, a video-assisted thoracoscopic lung biopsy should be considered to confirm a definitive diagnosis of OP [13]. Relapse of RIOP is common once tapering of steroid therapy begins. The optimal tapering schedule to avoid RIOP relapse is not yet known.

## 5. Conclusions

In summary, RIOP is an OP observed after radiotherapy involving a limited volume of lung. It is relatively rare, but its prognosis is good. Steroid treatment works well, but is related to relapses. Patient management should be symptom-oriented, and use of steroid should be limited to those who require rapid symptom relief.

## Abbreviations

RIOP	Radiation-induced organizing pneumonia
OP	Organizing pneumonia
COP	Cryptogenic organizing pneumonia
SOP	Secondary organizing pneumonia
BOOP	Bronchiolitis obliterans organizing pneumonia
SBRT	Stereotactic body radiotherapy
CT	Computed tomography
BAL	Bronchoalveolar lavage
CEP	Chronic eosinophilic pneumonia
ATM	Ataxia telangiectasia mutated
TGF-β	Transforming growth factor-β

## References

[1] American Thoracic Society/European Respiratory Society International Multidisciplinary Consensus Classification of the Idiopathic Interstitial Pneumonias (2002). This joint statement of the American Thoracic Society (ATS), and the European Respiratory Society (ERS) was adopted by the ATS board of directors, June 2001 and by the ERS Executive Committee, June 2001. Am. J. Respir. Crit. Care Med..

[2] Vasu T.S., Cavallazzi R., Hirani A., Sharma D., Weibel S.B., Kane G.C. (2009). Clinical and radiologic distinctions between secondary bronchiolitis obliterans organizing pneumonia and cryptogenic organizing pneumonia. Respir. Care.

[3] Crestani B., Kambouchner M., Soler P., Crequit J., Brauner M., Battesti J.P., Valeyre D. (1995). Migratory bronchiolitis obliterans organizing pneumonia after unilateral radiation therapy for breast carcinoma. Eur. Respir. J..

[4] Bayle J.Y., Nesme P., Bejui-Thivolet F., Loire R., Guerin J.C., Cordier J.F. (1995). Migratory organizing pneumonitis “primed” by radiation therapy. Eur. Respir. J..

[5] Otani K., Nishiyama K., Ito Y., Kawaguchi Y., Inaji H. (2014). Steroid treatment increases the recurrence of radiation-induced organizing pneumonia after breast-conserving therapy. Cancer Med..

[6] Takigawa N., Segawa Y., Saeki T., Kataoka M., Ida M., Kishino D., Fujiwara K., Ohsumi S., Eguchi K., Takashima S. (2000). Bronchiolitis obliterans organizing pneumonia syndrome in breast-conserving therapy for early breast cancer: Radiation-induced lung toxicity. Int. J. Radiat. Oncol. Biol. Phys..

[7] Miwa S., Morita S., Suda T., Suzuki K., Hayakawa H., Chida K., Nakamura H. (2004). The incidence and clinical characteristics of bronchiolitis obliterans organizing pneumonia syndrome after radiation therapy for breast cancer. Sarcoidosis Vasc. Diffuse Lung Dis..

[8] Ogo E., Komaki R., Fujimoto K., Uchida M., Abe T., Nakamura K., Mitsumori M., Sekiguchi K., Kaneyasu Y., Hayabuchi N. (2008). A survey of radiation-induced bronchiolitis obliterans organizing pneumonia syndrome after breast-conserving therapy in Japan. Int. J. Radiat. Oncol. Biol. Phys..

[9] Kubo A., Osaki K., Kawanaka T., Furutani S., Ikushima H., Nishitani H. (2009). Risk factors for radiation pneumonitis caused by whole breast irradiation following breast-conserving surgery. J. Med. Investig..

[10] Ogo E., Komaki R., Abe T., Uchida M., Fujimoto K., Suzuki G., Tsuji C., Suefuji H., Etou H., Hattori C. (2010). The clinical characteristics and non-steroidal treatment for radiation-induced bronchiolitis obliterans organizing pneumonia syndrome after breast-conserving therapy. Radiother. Oncol..

[11] Katayama N., Sato S., Katsui K., Takemoto M., Tsuda T., Yoshida A., Morito T., Nakagawa T., Mizuta A., Waki T. (2009). Analysis of factors associated with radiation-induced bronchiolitis obliterans organizing pneumonia syndrome after breast-conserving therapy. Int. J. Radiat. Oncol. Biol. Phys..

[12] Okada Y., Sakamoto S., Abe T., Shinozaki M., Gomi H., Kanemaki Y., Matsuoka S., Nakajima Y. (2015). Factors Predicting the Relapse of Radiation-Induced Organizing Pneumonia after Breast-Conserving Therapy. Open J. Radiol..

[13] Epler G.R., Kelly E.M. (2014). Systematic review of postradiotherapy bronchiolitis obliterans organizing pneumonia in women with breast cancer. Oncologist.

[14] Epler G.R. (2011). Bronchiolitis obliterans organizing pneumonia, 25 years: A variety of causes, but what are the treatment options?. Expert Rev. Respir. Med..

[15] Cohen A.J., King T.E., Downey G.P. (1994). Rapidly progressive bronchiolitis obliterans with organizing pneumonia. Am. J. Respir. Crit. Care Med..

[16] Lohr R.H., Boland B.J., Douglas W.W., Dockrell D.H., Colby T.V., Swensen S.J., Wollan P.C., Silverstein M.D. (1997). Organizing pneumonia: Features and prognosis of cryptogenic, secondary, and focal variants. Arch. Intern. Med..

[17] Crestani B., Valeyre D., Roden S., Wallaert B., Dalphin J.C., Cordier J.F. (1998). Bronchiolitis obliterans organizing pneumonia syndrome primed by radiation therapy to the breast. The Groupe d’Etudes et de Recherche sur les Maladies Orphelines Pulmonaires (GERM”O”P). Am. J. Respir. Crit. Care Med..

[18] Stover D.E., Milite F., Zakowski M. (2001). A newly recognized syndrome—Radiation-related bronchiolitis obliterans and organizing pneumonia. A case report and literature review. Respiration.

[19] Arbetter K.R., Prakash U.B., Tazelaar H.D., Douglas W.W. (1999). Radiation-induced pneumonitis in the “nonirradiated” lung. Mayo Clin. Proc..

[20] Van Laar J.M., Holscher H.C., van Krieken J.H., Stolk J. (1997). Bronchiolitis obliterans organizing pneumonia after adjuvant radiotherapy for breast carcinoma. Respir. Med..

[21] Hamanishi T., Morimatu T., Oida K., Kori Y., Taguchi Y., Tanaka E., Inoue T., Kato T., Maniwa K., Kobashi Y. (2001). Occurrence of BOOP outside radiation field after radiation therapy for small cell lung cancer. Nihon Kokyuki Gakkai Zasshi.

[22] Iijima M., Sakahara H. (2003). Radiation pneumonitis resembling bronchiolitis obliterans organizing pneumonia after postoperative irradiation for lung cancer: A case report. Nihon Igaku Hoshasen Gakkai Zasshi.

[23] Kwok E., Chan C.K. (1998). Corticosteroids and azathioprine do not prevent radiation-induced lung injury. Can. Respir. J..

[24] Nogi S., Nakayama H., Tajima Y., Okubo M., Mikami R., Sugahara S., Akata S., Tokuuye K. (2014). Cryptogenic organizing pneumonia associated with radiation: A report of two cases. Oncol. Lett..

[25] Falcinelli L., Bellavita R., Rebonato A., Chiari R., Vannucci J., Puma F., Aristei C. (2015). Bronchiolitis obliterans organizing pneumonia after radiation therapy for lung cancer: A case report. Tumori.

[26] Murai T., Shibamoto Y., Nishiyama T., Baba F., Miyakawa A., Ayakawa S., Ogino H., Otsuka S., Iwata H. (2012). Organizing pneumonia after stereotactic ablative radiotherapy of the lung. Radiat. Oncol..

[27] Ochiai S., Nomoto Y., Yamashita Y., Murashima S., Hasegawa D., Kurobe Y., Toyomasu Y., Kawamura T., Takada A., Noriko I. (2015). Radiation-induced organizing pneumonia after stereotactic body radiotherapy for lung tumor. J. Radiat. Res..

[28] Krengli M., Sacco M., Loi G., Masini L., Ferrante D., Gambaro G., Ronco M., Magnani C., Carriero A. (2008). Pulmonary changes after radiotherapy for conservative treatment of breast cancer: A prospective study. Int. J. Radiat. Oncol. Biol. Phys..

[29] Cordier J.-F. (2006). Cryptogenic organising pneumonia. Eur. Respir. J..

[30] Majori M., Poletti V., Curti A., Corradi M., Falcone F., Pesci A. (2000). Bronchoalveolar lavage in bronchiolitis obliterans organizing pneumonia primed by radiation therapy to the breast. J. Allergy Clin. Immunol..

[31] Cottin V., Frognier R., Monnot H., Levy A., DeVuyst P., Cordier J.F. (2004). Chronic eosinophilic pneumonia after radiation therapy for breast cancer. Eur. Respir. J..

[32] Cottin V., Cordier J.-F. (2012). Cryptogenic organizing pneumonia. Semin. Respir. Crit. Care Med..

[33] Roberton B.J., Hansell D.M. (2011). Organizing pneumonia: A kaleidoscope of concepts and morphologies. Eur. Radiol..

[34] Beardsley B., Rassl D. (2013). Fibrosing organising pneumonia. J. Clin. Path..

[35] Izykowski N., Kuehnel M., Hussein K., Mitschke K., Gunn M., Janciauskiene S., Haverich A., Warnecke G., Laenger F., Maus U. (2016). Organizing pneumonia in mice and men. J. Transl. Med..

[36] Endlich B., Radford I.R., Forrester H.B., Dewey W.C. (2000). Computerized video time-lapse microscopy studies of ionizing radiation-induced rapid-interphase and mitosis-related apoptosis in lymphoid cells. Radiat. Res..

[37] Cordier J.-F., Cottin V., Lazor R., Stoppa-Lyonnet D. (2016). Monoallelic germline ATM mutation and organising pneumonia induced by radiation therapy to the breast. Eur. Respir. J..

[38] Lee J., Paull T. (2007). Activation and regulation of ATM kinase activity in response to DNA double-strand breaks. Oncogene.

[39] Thompson D., Duedal S., Kirner J., McGuffog L., Last J., Reiman A., Byrd P., Taylor M., Easton D.F. (2005). Cancer risks and mortality in heterozygous ATM mutation carriers. J. Natl. Cancer Inst..

[40] Renwick A., Thompson D., Seal S., Kelly P., Chagtai T., Ahmed M., North B., Jayatilake H., Barfoot R., Spanova K. (2006). ATM mutations that cause ataxia-telangiectasia are breast cancer susceptibility alleles. Nat. Genet..

[41] Murofushi K.N., Oguchi M., Gosho M., Kozuka T., Sakurai H. (2015). Radiation-induced bronchiolitis obliterans organizing pneumonia (BOOP) syndrome in breast cancer patients is associated with age. Radiat. Oncol..

[42] Colletta A., Wakefield L., Howell F., Van Roozendaal K., Danielpour D., Ebbs S., Sporn M., Baum M. (1990). Anti-oestrogens induce the secretion of active transforming growth factor β from human fetal fibroblasts. Br. J. Cancer.

[43] Bentzen S.M., Skoczylas J.Z., Overgaard M., Overgaard J. (1996). Radiotherapy-related lung fibrosis enhanced by tamoxifen. J. Natl. Cancer Inst..

[44] Huang E.-Y., Wang C.-J., Chen H.-C., Sun L.-M., Fang F.-M., Yeh S.-A., Hsu H.-C., Hsiung C.-Y., Wu J.-M. (2000). Multivariate analysis of pulmonary fibrosis after electron beam irradiation for postmastectomy chest wall and regional lymphatics: Evidence for non-dosimetric factors. Radiother. Oncol..

[45] Harris E.E., Christensen V.J., Hwang W.-T., Fox K., Solin L.J. (2005). Impact of concurrent versus sequential tamoxifen with radiation therapy in early-stage breast cancer patients undergoing breast conservation treatment. J. Clin. Oncol..

[46] Pierce L.J., Hutchins L.F., Green S.R., Lew D.L., Gralow J.R., Livingston R.B., Osborne C.K., Albain K.S. (2005). Sequencing of tamoxifen and radiotherapy after breast-conserving surgery in early-stage breast cancer. J. Clin. Oncol..

[47] Azria D., Belkacemi Y., Romieu G., Gourgou S., Gutowski M., Zaman K., Moscardo C.L., Lemanski C., Coelho M., Rosenstein B. (2010). Concurrent or sequential adjuvant letrozole and radiotherapy after conservative surgery for early-stage breast cancer (CO-HO-RT): A phase 2 randomised trial. Lancet Oncol..

[48] McEvoy C.E., Niewoehner D.E. (1997). Adverse effects of corticosteroid therapy for COPD: A critical review. Chest.

[49] Rutz H.P. (2002). Effects of corticosteroid use on treatment of solid tumours. Lancet.

[50] Vento S., Cainelli F., Temesgen Z. (2008). Lung infections after cancer chemotherapy. Lancet Oncol..

[51] Herr I., Pfitzenmaier J. (2006). Glucocorticoid use in prostate cancer and other solid tumours: Implications for effectiveness of cytotoxic treatment and metastases. Lancet Oncol..

[52] Zhang C., Kolb A., Mattern J., Gassler N., Wenger T., Herzer K., Debatin K., Büchler M., Friess H., Rittgen W. (2006). Dexamethasone desensitizes hepatocellular and colorectal tumours toward cytotoxic therapy. Cancer Lett..

[53] Johkoh T., Muller N.L., Cartier Y., Kavanagh P.V., Hartman T.E., Akira M., Ichikado K., Ando M., Nakamura H. (1999). Idiopathic Interstitial Pneumonias: Diagnostic Accuracy of Thin-Section CT in 129 Patients. Radiology.

